# Comparative effects of estradiol and daidzein on the expression of endometrial cancer-related genes and histopathological parameters in the uterus of ovariectomized rats

**DOI:** 10.22038/ijbms.2025.89681.19349

**Published:** 2026

**Authors:** Vahid Setayesh, Asma Neisy, Maryam Niknam, Zahra Khoshdel, Farhad Koohpeyma, Sanaz Alaee, Fatemeh Zal

**Affiliations:** 1 Department of Biochemistry, School of Medicine, Shiraz University of Medical Science, Shiraz, Iran; 2 Research committee, Endocrine and Metabolism Research Center, Shiraz University of Medical Sciences, Shiraz, Iran; 3 Department of Reproductive Biology, School of Advanced Medical Sciences and Technologies, Shiraz University of Medical Sciences, Shiraz, Iran; 4 Department of Natural Sciences, West Kazakhstan Marat Ospanov Medical University, Aktobe, 030012, Kazakhstan; 5 Infertility Research Center, Shiraz University of Medical Sciences, Shiraz, Iran

**Keywords:** Endometrial neoplasms, Estrogens, Ovariectomy, Oxidative stress, Phytoestrogens, Rats

## Abstract

**Objective(s):**

This study compared the effects of daidzein (DZD) and 17-β-estradiol (E2) on uterine histopathology, expression of endometrial cancer-related genes, and anti-oxidant status in ovariectomized (OVX) rats.

**Materials and Methods:**

Thirty rats were divided into five groups (n=6): Sham, OVX, OVX+E2 (10 μg/kg/day), OVX+DZD (20 mg/kg/day), and DZD-only. After 50 days of treatment, uterine tissues were analyzed for histopathological changes, mRNA expression of ERα, ERβ, PTEN, EZH2, and Ki67, and oxidative stress markers (TAC, SOD, CAT, and MDA).

**Results:**

Ovariectomy induced endometrial atrophy, significantly downregulated the expression of all target genes (ERα, ERβ, PTEN, EZH2, and Ki67), decreased SOD and CAT activity and TAC level, and increased MDA. E2 treatment reversed these changes but induced hyperplastic effects. DZD administration significantly increased CAT and SOD activity and elevated ERβ and Ki67 expression compared with the OVX group. Crucially, DZD prevented uterine atrophy without inducing hyperplasia.

**Conclusion:**

DZD demonstrated a potentially beneficial effect by improving uterine anti-oxidant capacity and preventing atrophy, but without the hyperplastic changes associated with estradiol. These findings suggest that DZD may be a safer alternative for managing hypoestrogenic conditions, warranting further investigation.

## Introduction

For many years, hormone replacement therapy (HRT) was considered the primary solution for managing estrogen deficiency. However, recent evidence clearly shows that long-term use of HRT can increase the risk of several cancers, including breast cancer and endometrial cancer (EC) (1). EC is one of the most common gynecological cancers, primarily affecting postmenopausal women using estrogen-containing HRT. Approximately 80% of EC cases exhibit estrogen receptor (ER) expression and are classified as type I or estrogen-dependent (2, 3). Type II, which constitutes 10-20% of all cases, is more aggressive and classified as the estrogen-independent type (4). It’s reported that, by activating the estrogen receptor α (ERα), E2 provides the primary proliferative signal in the uterine epithelium, and long-term exposure is a significant risk factor for the development of EC, as seen with HRT (1, 5).

Phosphatase and Tensin homolog (PTEN) is a crucial tumor suppressor protein that plays a significant role in inhibiting cell proliferation (6). The most common genetic alteration observed in EC is a Loss of heterozygosity of chromosome 10q, where PTEN is located, or an intragenic mutation of PTEN; Thus, the loss of PTEN expression in endometrial hyperplasia may serve as an early indicator of an increased risk of EC. In a study by Mutter *et al*., PTEN mutations were found in 83% of endometrial adenocarcinomas and 55% of precursor lesions, while no mutations were detected in normal endometrial tissue (7, 8). It is important to note that PTEN mutations are rare in estrogen-independent forms of endometrial cancer. Furthermore, fluctuations in PTEN across a normal menstrual cycle suggest a potential link between estrogen signaling pathways and PTEN-related mechanisms. (9).

Enhancer of Zeste Homolog 2 (EZH2) is a specific methyltransferase and plays a crucial role in cancer progression. The EZH2 promoter contains several estrogen-responsive elements. (10). Thus, E2 could stimulate its expression in the uterus of both adult and young mice, as well as in ovariectomized mice (11). EZH2 expression is elevated in endometrial cancer (12) and other uterine abnormalities, such as fibroids (13), endometriosis, and uterine hyperplasia (11). EZH2 can enhance the proliferation and invasion of endometrial cancer cells (12). A more recent study found that inhibiting EZH2 expression significantly suppressed EC cell proliferation and invasion (14). 

Antigen Kiel 67 (Ki67) levels are frequently used as an indicator of cell proliferation and cell division (15). Ki67 can be an important prognostic indicator in endometrial cancer (16). Increased Ki67 expression has been reported in 40% to 80% of simple to complex hyperplasia and 100% of endometrial cancer cases. Therefore, Ki67 expression may serve as a marker for endometrial carcinogenesis and tumor cell proliferation (17).

Recent studies have shown that oxidative stress plays a remarkable role in various pathological conditions, including degenerative diseases, aging, reproductive disorders, and cancer (18, 19). Specifically, oxidative stress can disrupt gene expression and influence cell proliferation and apoptosis. The connection between oxidative stress and endometrial cancer has been established (20). Punnonen *et al*. found that superoxide dismutase (SOD) activity is significantly lower in cancerous tissues than in normal endometrial tissues (21), indicating that EC is associated with a compromised enzymatic anti-oxidant defense system. Furthermore, studies suggest that women with endometrial hyperplasia are exposed to increased levels of oxidative stress, and it appears their anti-oxidant defense systems are disrupted in the early stages of endometrial cancer (22-24).

Plant-derived substances, such as phytoestrogens, flavonoids, and alkaloids, have shown promising benefits in managing various gynecological-related conditions (25-27). The isoflavone daidzein (DZD) is a major phytoestrogen obtained from soybeans; its chemical structure resembles that of estrogen, allowing it to bind to estrogen receptors (28). Several studies have demonstrated that DZD and its metabolites can help reduce menopausal symptoms in women, such as hot flashes and muscle and joint pain (29)ÿÿÿ. As a result, DZD may be a potential therapeutic option for managing complications associated with estrogen deficiency. Several studies have explored the impacts of isoflavones on the endometrium. Puerarin and parthenolide, both flavonoids, have been shown to inhibit the proliferation of ovarian endometrioma (OE) cells (30). However, the impact of DZD on endometrial cancer-related markers in comparison to E2 remains unclear. Therefore, in this study, we investigated the effects of 20 mg/kg/day DZD on histopathological changes, oxidant and anti-oxidant status, and endometrial cancer-related genes in the uterus of ovariectomized rats compared to 17-β estradiol. 

## Materials and Methods

### Study type and ethical approval for animal use

Ethical approval was obtained from the Ethics Committee of Shiraz University of Medical Sciences (IR.SUMS.AEC.1403.036), and all procedures were conducted in accordance with the National Institutes of Health (NIH) guidelines for the care and use of laboratory animals.

### Animal care and handling

A total of 30 adult female Sprague-Dawley rats were housed at the Center for Comparative and Experimental Medicine at Shiraz University of Medical Sciences in Shiraz, Iran. The rats, which weighed between 230 g and 250 g, exhibited at least three consecutive regular estrous cycles. They were kept in eight polypropylene cages under controlled environmental conditions, including 23 ± 2 °C, 40 ± 5% humidity, and a 12-hour light/dark cycle. The rats were provided with a standard pelleted diet (Pars Dam, Tehran, Iran) and had access to water ad libitum.

### Experimental design

Two weeks after ovariectomy, the rats were randomly assigned into five experimental groups (n=6 per group) as detailed in Table 1. Briefly, the groups were: Sham+Vehicle: Sham-operated rats receiving the vehicle via oral gavage. OVX+DZD: Ovariectomized rats receiving DZD via oral gavage (31). OVX+Vehicle: Ovariectomized rats receiving the vehicle via oral gavage.OVX+E2: Ovariectomized rats receiving 17β-estradiol (E2) via subcutaneous (S.C.) injection (32, 33). DZD+Vehicle: Sham-operated rats receiving DZD via oral gavage. The vehicle (90% corn oil and 10% ethanol) was used for treatments (34). DZD and its corresponding vehicle were administered by oral gavage. 17β-Estradiol (E2) was administered by subcutaneous (S.C.) injection. All treatments were administered once daily for 50 consecutive days. The dose selection was based on previous studies. 

All groups received their respective treatments once daily for 50 days. At the end of the experimental period, the rats were euthanized, and the uterus was excised and divided into three parts. The first part was used for histopathological examination, the second for gene expression analysis, and the third was homogenized in phosphate-buffered saline (PBS, pH 7.4) and centrifuged at 4,000 × g for 40 min at 4 °C. The supernatant was stored at −80 °C for further analysis. The supernatants were used to measure total protein, malondialdehyde (MDA), total anti-oxidant capacity (TAC), and the enzymatic activities of superoxide dismutase (SOD) and catalase (CAT).

### Ovariectomy procedure

At the first step, the rats were anesthetized with an intraperitoneal injection of 10% ketamine (0.08 mL/100 g body weight; RompunVR/SP/Brazil) and 2% xylazine (0.04 mL; KetalarVR/SP/Brazil). The dorsal area of the animals was shaved and cleaned with 70% ethanol. A single incision, measuring 2 cm in length, was made under sterile conditions in the lower abdominal region between the umbilicus and the pubis. The abdominal muscles and skin were then opened, allowing for the removal of both ovaries. Next, 1-2 ml of physiological saline solution was poured into the abdominal cavity. The incision was subsequently sutured closed, and lidocaine, along with tetracycline ointments, was applied locally to the incision site (35). The anestrous state in OVX rats and the regular estrous cycle were confirmed via vaginal swabs. 

### Histopathological examination of uterus tissue

A small piece of uterine tissue was fixed in 10% neutral buffered formalin at 4 °C for over 48 hr. The tissues were then dehydrated, embedded in paraffin, sectioned, and stained with hematoxylin and eosin (H&E) for histopathological analysis (36).

### Estimation of the volume density ratio of the uterus structures

**To assess the volume density ratio (Vv) of the histological structures, such as primetriume, myometrium, gland, and endometrium, we employed the method of point-counting and Delesse’s formula:**

$v_{v\left(\mathrm{structure}\right)=}\sum_{i=1}^{n}p(\mathrm{structure})/\sum_{i=1}^{n}p(\mathrm{reference})$

**In this formula, “ΣP structure” represents the total number of points that intersect with the favored structure within the uterus. At the same time, “ΣP references” indicates the total number of points that hit the uterus section for each animal in the study. To estimate the thickness of the different layers of the uterus, a probe with parallel lines and equal distances (Orthogonal Intercepts Probe) was used. Therefore, to estimate the thickness of the layers, the desired probe was placed on the tissue. Then, for each point where the probe lines collided with the surface of the desired layer, the shortest linear path perpendicular to the other surface of the layer was drawn, and its length was measured. However, since the thicknesses in different parts of the desired layer may differ, the following formula was used to eliminate this error: T = π/4 × L**_0_ **. T indicates the estimated thickness, and L0 represents the average measured thickness (** 37 **).**

### Gene expression analysis

*Uterine RNA extraction and cDNA synthesis*

Total RNA was extracted from uterine tissue using RiboEx solution (GeneAll Biotechnology Company, Seoul, Korea). The quality and purity of the extracted RNA were evaluated using 1.5% w/v agarose/TEA gel electrophoresis and Nanodrop, respectively. cDNA was synthesized using the Sina Clone cDNA synthesis kit (Tehran, Iran).

***Reverse transcription and real-time quantitative PCR (RT-qPCR) analysis***

RT-qPCR was conducted in 96-well plates using Low Rox RealQ Plus 2x Master Mix Green (Ampliqon A/S, Denmark) on a QuantStudio3 instrument (Applied Biosystems, Carlsbad, CA). The RT-qPCR protocol included an initial denaturation step at 95 °C for 10 min, followed by 40 cycles of 30 seconds at 95 °C, 30 seconds at 60 °C, and 30 seconds at 72 °C, with a final extension at 72 °C for 10 min. The mRNA expression levels of target genes (ERα, ERβ, PTEN, EZH2, and Ki67) were normalized to β-actin, which was validated as a stable reference gene, as its Ct values did not vary significantly across the different experimental groups (*P*>0.05, Kruskal-Wallis test). Data were analyzed and reported using the ΔΔCT method. The sequences of all primers used, along with their amplicon sizes and calculated efficiencies, are listed in Supplementary Table S1. Representative melt curves and standard curves are provided in Supplementary Figures S1 and S2, respectively. The calculated efficiencies for all primer pairs were within the optimal range of 90–110%, allowing the use of the ΔΔCT method without efficiency correction.

### Evaluation of oxidative stress status

*Determination of SOD activity*

The activity of superoxide dismutase (SOD) in the supernatants of the uterus homogenate was evaluated using a commercial kit (Kiazist, KSOD-96, Hamedan, Iran) following the manufacturer’s instructions. This assay relies on the conversion of purple Resazurin to pink Resorufin in the presence of superoxide anions (O₂⁻), inhibited by SOD. The intensity of the resulting color, which correlates with SOD activity, was measured at 540 nm using a spectrophotometer.

### Determination of CAT activity

Catalase (CAT) activity was measured using the procedure of Aebi (38) with minor modifications. Briefly, 95 µl of 30 mM H₂O₂ was added to a cuvette containing 60 µl of supernatant of uterus homogenate, 130 µl of phosphate buffer, and 325 µl of distilled water. The decomposition of H₂O₂ was monitored by measuring the decrease in absorbance at 240 nm. CAT activity in the clear supernatant of the uterus homogenate was calculated as mmol of H₂O₂ consumed per minute per mg of tissue protein, using a molar extinction coefficient of 43.60 l/mol/cm for H₂O₂.

### Determination of TAC level

Total Anti-oxidant Capacity (TAC) was measured using the manufacturer’s protocol (Kiazist, KTAC96-96, Hamedan, Iran). This method, known as the CUPRAC assay (Cupric Reducing Anti-oxidant Capacity), measures the reduction of cupric ions (Cu²⁺) to cuprous ions (Cu⁺) in the presence of anti-oxidants. The reaction produces a colored complex when combined with a chromogen, and the intensity of this color, measured spectrophotometrically at 450 nm, is proportional to the sample’s anti-oxidant capacity.

### Determination of MDA level

MDA, a marker of lipid peroxidation, was measured using a colorimetric assay following an established protocol (39).  In brief, 0.5 ml of uterine homogenate supernatant was combined with 2 ml of thiobarbituric acid (TBA) reagent, which contained 0.375% TBA, 15% trichloroacetic acid, and 0.25 mol/l HCl. The mixture was heated in a water bath at 95 °C for 30 min, then rapidly cooled and centrifuged at 8000 × g for 15 min at 4 °C. The absorbance of the pink-colored supernatant was measured at 532 nm. MDA concentration was calculated using tetraethoxypropane (TEP) as a standard, and results were expressed as nmol/mg protein.

### Protein concentration assay

Protein concentrations in the uterine homogenate supernatant were quantified using the Bradford method, with bovine serum albumin (BSA) as the standard (40).

### Statistical analysis

Statistical analyses were conducted using GraphPad Prism 10 (GraphPad Software, Inc., San Diego, CA, USA), and data are presented as mean ± standard deviation (SD). The normality of all data sets was assessed using the Shapiro-Wilk test. Since the data were not normally distributed, group comparisons were performed using the non-parametric Kruskal-Wallis test, followed by Dunn’s post hoc test for pairwise comparisons. A p-value of less than 0.05 (*P*<0.05) was considered statistically significant.

## Results

### Histopathological analysis

*The histoarchitecture of uterine tissue*

**Figure 1:**
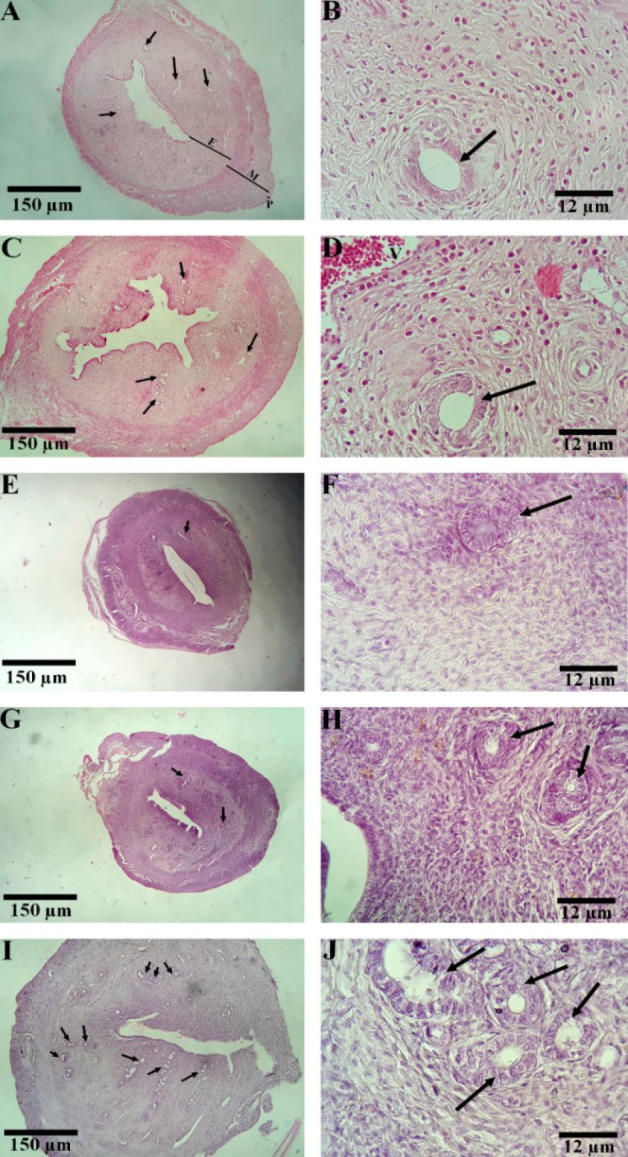
Histoarchitecture of uterine tissue

The Sham and DZD groups exhibited normal stromal density, typical gland count, and morphology. The glands were tubular and were distributed uniformly within the abundant stroma. In the OVX group, the number of glands was significantly reduced compared to the sham group (*P*<0.05). Fifty constitutive days of treatment with DZD increased the number of endometrial glands compared to the OVX group (*P*<0.05). The glands were scattered throughout the abundant stroma and retained their tubular morphology. Administration of E2 markedly increased the gland count, reduced the spacing between glands, and preserved their tubular morphology compared to the OVX rats (*P*<0.05) (Figure 1).

*The volume of the endometrial tissue*

**Figure 2:**
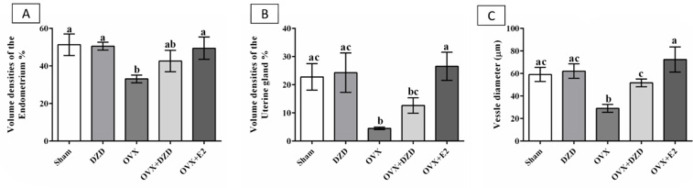
The volume of uterine-related structures in ovariectomized rats

The endometrial volume in the OVX group decreased by 36% compared to the sham group (*P*<0.05). However, the differences between other groups and the sham group were not statistically significant. The comparison between groups revealed that estradiol (E2) increased endometrial volume by 48% in the OVX+E2 group compared with the OVX group (*P*<0.05). Additionally, DZD administration increased endometrial volume by 20% in the OVX+DZD group compared with the OVX group, although this difference was not statistically significant. There was no significant difference in endometrial volume between the OVX+DZD and OVX+E2 groups (Figure 2).

*The endometrial vessels and glandular layer*

The glandular volume in the OVX group decreased by 82% compared to the sham group (*P*<0.05). Estradiol caused a 5.8-fold increase in glandular volume in the OVX+E2 group compared to the OVX group (*P*<0.05). Daidzein induced a 2.8-fold increase in glandular volume in the OVX+DZD group compared to the OVX group, although this difference was not statistically significant. Estradiol resulted in a 2-fold increase in glandular volume in the OVX+E2 group compared to the OVX+DZD group (*P*<0.05).

The vascular diameter in the OVX group decreased by 50% compared to the sham group (*P*<0.05). Estradiol caused a 2.4-fold increase in vascular diameter in the OVX+E2 group compared to the OVX group (*P*<0.05). Similarly, DZD led to a 1.25-fold increase in vascular diameter in the OVX+DZD group compared to the OVX group (*P*<0.05). Estradiol resulted in a 1.4-fold increase in vascular diameter in the OVX+E2 group compared to the OVX+DZD group (*P*<0.05) (Figure 2).

*The thickness of the uterine layers*

The OVX group exhibited significant reductions in endometrial, perimetrial, and myometrial thickness compared to the sham group (*P*<0.05). Specifically, endometrial thickness decreased by 70%, perimetrial thickness by 55%, and myometrial thickness by 50%. In the OVX+DZD group, endometrial and myometrial thicknesses were reduced by 50% and 30%, respectively, compared to the sham group (*P*<0.05).

Estradiol (E2) significantly increased thickness in all measured parameters: endometrial thickness increased by 3.6-fold, perimetrial thickness by 1.9-fold, and myometrial thickness by 2-fold in the OVX+E2 group compared to the OVX group (*P*<0.05). DZD also showed a positive but non-significant effect, inducing a 1.8-fold increase in endometrial thickness, a 1.5-fold increase in perimetrial thickness, and a 1.35-fold increase in myometrial thickness in the OVX+DZD group compared to the OVX group.

**Figure 3:**
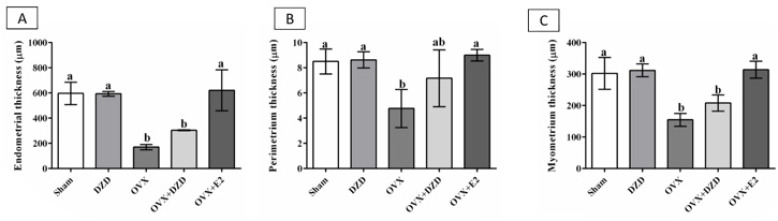
The thickness of uterine layers in ovariectomized rats

Notably, there were no significant differences in endometrial, perimetrial, or myometrial thickness between the OVX+DZD and OVX+E2 groups, suggesting that DZD partially mimics the effects of estradiol on uterine tissue (Figure 3).

### Gene expression results

*Effect of estradiol and daidzein on ERα gene expression in the uterus*

**Figure 4:**
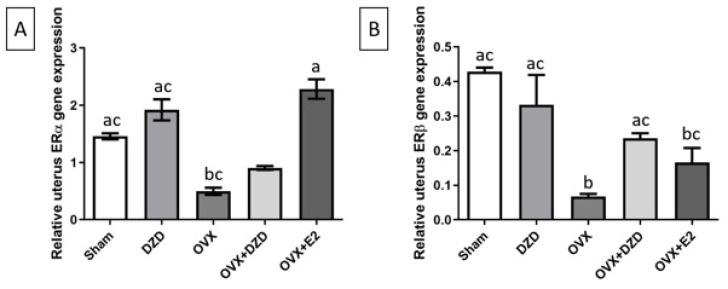
Effect of estradiol and daidzein on ERα (A) and ERβ (B) gene expression in the uterus of ovariectomized rats

 As shown in Figure 4, ERα gene expression was significantly reduced in the OVX group compared to the Sham group, with a decrease of over 65% (*P*<0.05). No significant differences in ERα expression were observed in the other groups compared to the Sham group. However, the DZD group exhibited a 1.3-fold increase in ERα expression compared to the OVX group. Similarly, the OVX+E2 group showed a 4.6-fold increase in ERα expression relative to the OVX group (*P*<0.05, **Cohen’s d = 4.3; see Supplementary Table S2 for full statistical details**). Although the OVX+DZD group did not show a statistically significant difference in ERα expression compared to the OVX group, there was an increase of over 50%. Notably, the OVX+E2 group demonstrated a more than 3-fold higher ERα expression compared to the OVX+DZD group (*P*<0.05).

*Effect of estradiol and daidzein on ERβ gene expression in the uterus*

As shown in Figure 4, the expression of the ERβ gene in the OVX group decreased by more than 80% compared to the Sham group (*P*<0.05). Similarly, compared to the Sham group, ERβ expression in the OVX+E2 group was reduced by over 60% (*P*<0.05). No significant differences in ERβ expression were observed between the OVX+DZD and DZD groups and the Sham group. However, ERβ expression in the OVX+DZD group was significantly higher than in the OVX group, showing a more than 3-fold increase (*P*<0.05). Additionally, ERβ expression in the OVX+DZD group was considerably higher than in the OVX+E2 group, with a 1.4-fold increase compared to the OVX+E2 group.

*Effect of estradiol and daidzein on PTEN gene expression in the uterus*

**Figure 5:**
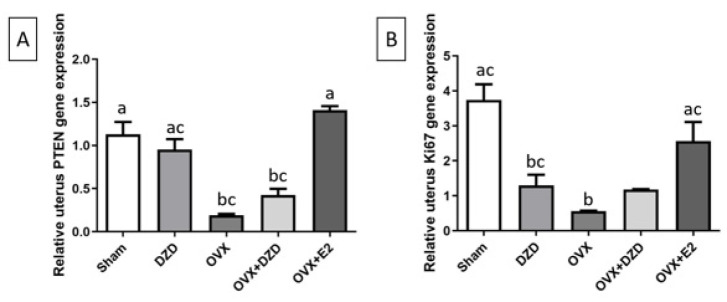
Effect of estradiol and daidzein on PTEN (A) and Ki67 (B) gene expression in the uterus of ovariectomized rats

As shown in Figure 5, PTEN expression in the uterus was significantly reduced by 81% in the OVX group compared with the Sham group (*P*<0.05). Similarly, PTEN expression in the OVX+DZD group decreased by more than 60% compared to the Sham group (*P*<0.05). Although there was no significant difference in PTEN expression between the OVX+DZD and OVX groups, PTEN expression in the OVX+DZD group was significantly higher than in the OVX+E2 group, where estrogen increased PTEN expression by more than 7-fold compared to the OVX group (*P*<0.05). Furthermore, PTEN expression in the OVX+E2 group was approximately 4-fold higher than in the OVX+DZD group (*P*<0.05).

*Effect of estradiol and daidzein on Ki67 gene expression in the uterus*

As shown in Figure 5, the expression of the Ki67 gene in the uterus was significantly reduced by 83% in the OVX group compared to the Sham group. Similarly, Ki67 expression in the DZD group decreased by more than 78% compared to the Sham group (*P*<0.05). However, no significant differences in Ki67 expression were observed between the OVX+DZD and OVX+E2 groups and the Sham group. Compared to the OVX group, Ki67 expression was significantly higher in the OVX+E2 group (*P*<0.05). Additionally, Ki67 expression in the OVX+DZD group was more than 2-fold higher than in the OVX group (*P*<0.05). Furthermore, Ki67 expression in the OVX+E2 group was approximately 1.5-fold higher than in the OVX+DZD group (*P*<0.05).

*Effect of estradiol and daidzein on EZH2 gene expression in the uterus*

**Figure 6:**
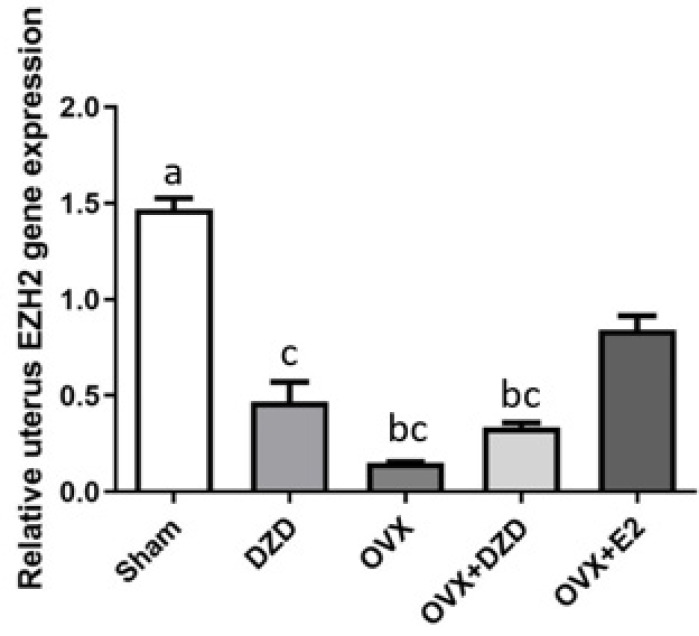
Effect of estradiol and daidzein on EZH2 gene expression in the uterus of ovariectomized rats

As shown in Figure 6, the expression of the EZH2 gene in the uterus was significantly reduced by 89% in the OVX group compared to the Sham group (*P*<0.05). Similarly, EZH2 expression in the DZD group decreased by more than 75% compared to the Sham group (*P*<0.05). The OVX+DZD group also showed a reduction of over 75% in EZH2 expression compared to the Sham group (*P*<0.05). In contrast, the OVX+E2 group showed no significant difference in EZH2 expression compared to the Sham group. Compared to the OVX group, EZH2 expression was significantly higher in the OVX+E2 group, with a 5-fold increase (*P*<0.05). Additionally, EZH2 expression in the OVX+E2 group was approximately 2.4-fold higher than in the OVX+DZD group (*P*<0.05).

### Oxidative stress status results

*Effect of daidzein on catalase-specific activity in rat uterine tissue*

**Figure 7:**
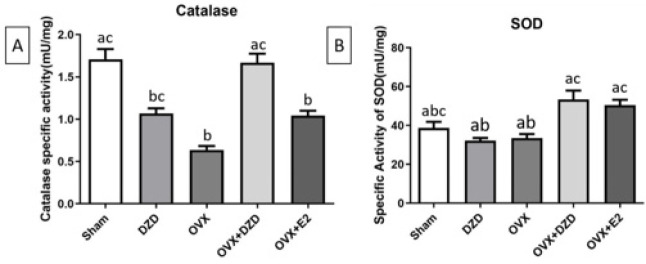
Effect of estradiol and daidzein treatment on the activity of CAT(A) and SOD(B) in uterus homogenate of experimental rats

As shown in Figure 7, the specific activity of catalase (CAT) was significantly higher in the OVX and OVX+E2 groups than in the Sham group. In the OVX+E2 group, catalase activity increased by nearly 40% compared to the Sham group, while in the OVX group, it decreased by more than 60% (*P*<0.05). Compared to the OVX group, estrogen treatment significantly increased catalase-specific activity, with a 65% increase in the OVX+E2 group (*P*<0.05). Similarly, treatment with DZD in the OVX+DZD group resulted in a more than 2.5-fold increase in catalase-specific activity compared to the OVX group **(**P<0.0**01, Cohen’s d = 3.6; see Supplementary Table S3 for full statistical details)**. Additionally, the difference in catalase-specific activity between the OVX+E2 and OVX+DZD groups was significant (*P*<0.05), with DZD treatment leading to a 1.5-fold higher enzyme activity compared to the estrogen-treated group.

*Effect of daidzein and estradiol administration on superoxide dismutase-specific activity in rat uterine tissue*

As shown in Figure 7, no significant differences in SOD-specific activity were observed between any of the experimental groups and the Sham group. However, the OVX+E2 group showed a significant increase in SOD-specific activity compared to the OVX group (*P*<0.05), with estrogen treatment increasing enzyme activity by more than 1.5-fold. Similarly, the OVX+DZD group exhibited a significant increase in SOD-specific activity compared to the OVX group (*P*<0.05), with DZD treatment increasing enzyme activity by approximately 1.6-fold. However, there was no significant difference in SOD-specific activity between the OVX+DZD and OVX+E2 groups.

*Effect of daidzein administration on MDA level in rat uterine tissue*

**Figure 8:**
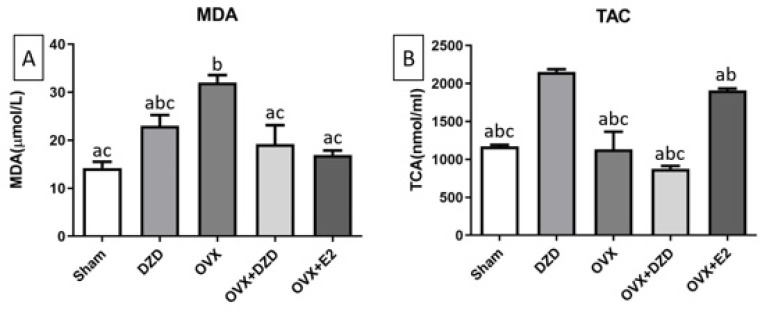
Effect of estradiol and daidzein treatment on the activity of MDA(A) and TAC(B) in uterus homogenate of experimental rats

As shown in Figure 8, MDA levels in the OVX group were significantly higher than in the Sham group, with a more than 2.2-fold increase in the OVX group (*P*<0.05). However, no significant differences in MDA levels were observed between the OVX+DZD and OVX+E2 groups compared to the Sham group. The most notable difference was between the OVX+DZD and OVX groups, in which DZD administration reduced MDA levels by more than 60% in the OVX+DZD group compared with the OVX group (*P*<0.05). Similarly, estrogen treatment significantly reduced MDA levels by over 50% in the OVX+E2 group compared to the OVX group (*P*<0.05). However, there was no significant difference in MDA levels between the OVX+E2 and OVX+DZD groups.

*Effect of daidzein on TAC level in rat uterine tissue*

As shown in Figure 8, the TAC in the OVX+E2 group was significantly higher than in the Sham group, with TAC levels approximately 1.6-fold greater in the OVX+E2 group (*P*<0.05). Similarly, the DZD group showed a significant increase in TAC compared to the Sham group, with levels approximately 1.8-fold higher (*P*<0.05). However, no significant differences in TAC were observed between the OVX+DZD and OVX groups compared to the Sham group. The OVX+E2 group exhibited a significantly higher TAC than the OVX group, with levels approximately 1.9-fold greater (*P*<0.05). In contrast, there was no significant difference in TAC between the OVX+DZD and OVX groups. Additionally, TAC levels in the OVX+E2 group were significantly higher than in the OVX+DZD group, with levels approximately 2-fold greater (*P*<0.05).

## Discussion

17-β estradiol is crucial for maintaining uterine health and function by interacting with estrogen receptors, particularly ERα (41). Estrogen deficiency conditions, such as menopause or primary ovarian insufficiency (POI), and inadequate levels of E2 lead to a suppressed estrogen receptor expression and reduction in the volume and thickness of the uterus, resulting in endometrial atrophy (42, 43), as shown by histological analysis of uterine tissue in ovariectomized rats. A normal proliferative uterus is typically characterized by tubular endometrial glands evenly distributed within a rich stroma (44). In simple hyperplasia, the glands exhibit variations in shape and size, appearing irregular but still present within the stroma. In contrast, hyperplasia is defined by compact glands and scattered stroma with indistinct nuclei (45, 46). Histopathological examination of the uterus in the OVX group showed that the number of glands in this group was significantly reduced compared with the sham group. Administration of DZD in the OVX+ DZD group increased the number of glands, indicating its ability to induce cell proliferation, but did not lead to hyperplasia. Our data showed that DZD treatment increased endometrial volume and thickness in OVX+DZD rats; however, it was less effective than E2. These results are consistent with those reported by Ishimi *et al*. (47). The difference in the effects of the two treatments may be attributed to the varying affinities of phytoestrogens and E2 for the estrogen receptors. Recent studies suggest that phytoestrogens bind more strongly to ERβ, while E2 is more effective at activating ERα, which is predominantly expressed in the uterus (48).

The proliferative effects of DZD on estrogen-sensitive tissues reported in the literature are complex and highly context-dependent, underscoring the significance of our findings. Our results showing that DZD prevents uterine atrophy without causing hyperplasia align with several *in vivo *studies. Importantly, a study directly comparing DZD to genistein in ovary-intact middle-aged rats found that DZD (35 mg/kg, s.c, 4 weeks) did not increase uterine wet weight or cause hyperplastic changes, while genistein led to significant hyperplasia (48). This matches a chronic feeding study in female rats, in which diets containing up to 1000 mg DZD per kg of feed caused no notable histomorphological damage in the uterus, ovary, or mammary tissue (49). However, it is also essential to recognize that other studies have reported stimulatory effects. For example, DZD has been shown to promote the growth of MCF-7 breast cancer cells in culture and enhance some estrogen responses in the immature rat uterus (50). The apparent contradiction is probably due to factors like dosage (supraphysiological versus physiological), metabolic conversion to equol, hormonal status of the model (ovariectomized versus immature versus ovary-intact), and the specific tissue examined.

An analysis of uterine ER gene expression revealed that treatment with E2 resulted in the highest expression levels of ERα and the greatest degree of uterine growth compared to the OVX and OVX+DZD groups. Although the impact of DZD on ERα expression compared to OVX+E2 or OVX rats was not statistically significant, DZD significantly increased ERβ gene expression, consistent with the study by Kim *et al*. (49). A significant concern regarding the use of phytoestrogens, such as genistein and DZD, is their potential adverse effects on reproductive organs, such as the development of endometrial cancer (50). In general, ERα levels are higher than ERβ in Endometrial cancer. In endometrial tissue, ERβ is crucial in maintaining normal homeostasis, cell cycling, and tissue remodeling (51). The lower level of ERα expression and the higher level of ERβ in OVX+DZD compared to OVX+E2 are clinically important, as they indicate that DZD administration does not induce strong proliferation and may be associated with a lower risk of endometrial cancer.

PTEN is likely the most extensively studied biomarker for endometrial cancer, as the PTEN gene is the most frequently mutated in this malignancy (52). Our data indicated that the changes in PTEN gene expression observed in the OVX+DZD group were not statistically significant compared with either the OVX rats or the OVX+E2 rats. Additionally, PTEN levels in the OVX+DZD group were lower than those in the sham group. This finding is consistent with the study by Aguilar *et al*., which demonstrated that scattered Pax2-positive glands and/or PTEN deficiency are common occurrences in normal endometrium (53). Since PTEN is not the only factor involved in endometrial carcinogenesis (54). Additional factors must be considered for accurate interpretation and conclusions. In this study, the favorable expression of other investigated genes and the overall condition of the uterine tissue further support the positive efficacy of DZD.

EZH2 promotes endometrial carcinoma proliferation and invasion, mediates epithelial-mesenchymal transition in endometriosis, and disrupts the expression of DNA repair genes in uterine fibroids (11, 55). Our data showed that estradiol significantly increased EZH2 expression in the uterus. However, this expression was significantly reduced following ovariectomy compared to treatment with DZD. Given the role of EZH2 in cell proliferation and cancer progression, it can be argued that long-term HRT might elevate the risk of endometrial cancer. Eskander *et al*. demonstrated that EZH2 expression was elevated in all types of endometrial cancer cell lines and human endometrial cancer tissue samples compared to controls. Therefore, DZD could be a safer alternative to estradiol for uterine tissue (11, 12).

Our results demonstrated that the absence of E2 in the OVX group led to uterine growth arrest and subsequent atrophy, which was associated with markedly low Ki67 levels. Ki67 expression significantly increased following E2 treatment in the OVX + E2 group. In contrast, administration of DZD resulted in a more modest increase in Ki67 levels and less pronounced endometrial growth, consistent with the histological findings. These data suggest that DZD may help prevent uterine atrophy without promoting excessive proliferative activity, thereby potentially reducing the risk of endometrial cancer. Furthermore, administering DZD to non-ovariectomized animals in the presence of endogenous estrogen decreased Ki67 expression and maintained a regular proliferative uterine pattern. Given that elevated Ki67 expression in patients with endometrial cancer correlates with lower survival rates and higher recurrence risk, our findings indicate that modulating Ki67 activity could impede cell cycle progression, highlighting it as a promising therapeutic target (15, 56).

Maintaining a balance between pro-oxidants and anti-oxidants is crucial for cellular integrity, as increased oxidative stress can damage lipids, proteins, and nucleic acids (57). Following menopause, reduced estrogen levels are associated with increased free radical production, decreased anti-oxidant activity, and elevated oxidative damage (41, 58). In line with this, our data showed that OVX rats exhibited a significant reduction in total anti-oxidant capacity, decreased SOD and catalase activity, and elevated MDA levels. Treatment with E2 in OVX + E2 rats essentially reversed these changes. Administration of DZD also increased SOD and catalase activity and reduced MDA levels compared with the OVX group; however, it did not significantly affect total anti-oxidant capacity (TAC). These findings suggest that DZD may partially mitigate oxidative stress in the uterus, but further studies are warranted to clarify its mechanisms and overall impact on anti-oxidant status.

A limitation of this study is that we assessed gene expression only at the mRNA level. Future work should include protein-level analysis (e.g., western blotting or immunohistochemistry) to confirm these findings and provide deeper mechanistic insight.

Hematoxylin and eosin staining (A, C, D, E, G and I) magnification 40× and (B, D, F, H, J) magnification 400× (A and B) sham group, (C and D) DZD group, (E and F) OVX group, (G and H) OVX + DZD group, (I and J) OVX + E2 group

Arrow marks indicate uterine glands. E: the endometrium, M: myometrium, P: perimetrium V: vessels

The Ovariectomies reduce the number of cells and glands in the uterus of the OVX rats. Estrogen and daidzein increase the number of cells and glands. In the OVX + DZD group, a normal proliferative uterus was observed, while in the OVX + E group, a hyperproliferative uterus with an increase in the number of glands was observed. Sham: Sham+Vehicle; DDZ: Daidzein+ Vehicle; OVX: Ovariectomy + Vehicle; OVX+DDZ: Ovariectomy+ daidzein; OVX+E2: Ovariectomy+ 17βestradiol

Volume density of Endometrial (A), volume density of glands (B), and vessel diameter(C). Significant differences (*P*<0.05) are shown with the sham group (a), with the OVX group (b), and with the OVX+DZD group (c).

Sham: Sham+Vehicle; DZD: Daidzein+ Vehicle; OVX: Ovariectomy + Vehicle; OVX+DZD: Ovariectomy+ daidzein; OVX+E2: Ovariectomy+ 17βestradiol

Endometrial thickness (A), Perimetrium thickness (B), and myometrium thickness (C). Significant differences (*P*<0.05) are shown with the sham group (a), with the OVX group (b), and with the OVX+DZD group (c)

Sham: Sham+Vehicle; DZD: Daidzein+ Vehicle; OVX: Ovariectomy + Vehicle; OVX+DZD: Ovariectomy+ daidzein; OVX+E2: Ovariectomy+ 17βestradiol

The results shown on this graph are reported as Mean ± SD; the columns represent the mean, and error bars represent SEM from 6 rats. ERα and ERβ gene expression levels were normalized to the internal control beta-actin. Significant differences (*P*<0.05) are shown with the sham group (a), with the OVX group (b), and with the OVX+DZD group (c). Sham: Sham+Vehicle; DZD: Daidzein+ Vehicle; OVX: Ovariectomy + Vehicle; OVX+DZD: Ovariectomy+ daidzein; OVX+E2: Ovariectomy+ 17βestradiol

The results shown on this graph are reported as Mean ± SD, and the columns represent the mean, and error bars represent SEM, from 6 rats. PTEN and Ki67gene expression levels were normalized to the internal control beta-actin. Significant differences (*P*<0.05) are shown with the sham group (a), with the OVX group (b), and with the OVX+DZD group (c)

Sham: Sham+Vehicle; DZD: Daidzein+ Vehicle; OVX: Ovariectomy + Vehicle; OVX+DZD: Ovariectomy+ daidzein; OVX+E2: Ovariectomy+ 17βestradiol

The results shown on this graph are reported as Mean ± SD, and the columns represent the mean, and error bars represent SEM, from 6 rats. EZH2 gene expression levels were normalized to the internal control beta-actin. Significant differences (*P*-Value <0.05) are shown with the sham group (a), with the OVX group (b), and with the OVX+DZD group (c).

Sham: Sham+Vehicle; DZD: Daidzein+ Vehicle; OVX: Ovariectomy + Vehicle; OVX+DZD: Ovariectomy+ daidzein; OVX+E2: Ovariectomy+ 17βestradiol

Data are presented as mean ± SEM. sample size (n) = 6, *P*<0.05. Significant differences (*P*<0.05) are shown with the sham group (a), with the OVX group (b), and with the OVX+DZD group (c).

Sham: Sham+Vehicle); DZD: Daidzein+ Vehicle; OVX: Ovariectomy + Vehicle; OVX+DZD: Ovariectomy+ daidzein; OVX+E2: Ovariectomy+ 17βestradiol

Data are presented as mean ± SEM. sample size (n) = 6, *P*<0.05. Significant differences (*P*<0.05) are shown with the sham group (a), with the OVX group (b), and with the OVX+DZD group (c).

Sham: Sham+Vehicle; DZD: Daidzein+ Vehicle; OVX: Ovariectomy + Vehicle; OVX+DZD: Ovariectomy+ daidzein; OVX+E2: Ovariectomy+ 17βestradiol.

## Conclusion

Estrogen deficiency can lead to physiological changes that negatively impact quality of life, highlighting the need to identify safe therapeutic approaches. In this study, DZD was found to modestly enhance the activity of anti-oxidant enzymes in the uterus, suggesting it may help maintain oxidative balance. Gene expression analyses related to endometrial hyperplasia and cancer indicated that DZD might help mitigate some effects of estrogen deficiency, without showing the pro-proliferative effects typically associated with estrogen therapy. However, further studies are needed to assess the long-term safety and efficacy of DZD.

## Data Availability

The data used in this study are available upon request.

## Declaration

The authors declare that no artificial intelligence (AI) tools or technologies were used in the preparation of this manuscript.

## References

[1] Lu JJ, Zhang X, Abudukeyoumu A, Lai ZZ, Hou DY, Wu JN (2023). Active estrogen-succinate metabolism promotes heme accumulation and increases the proliferative and invasive potential of endometrial cancer cells. Biomolecules.

[2] Emons G, Fleckenstein G, Hinney B, Huschmand A, Heyl W (2000). Hormonal interactions in endometrial cancer. Endocr Relat Cancer.

[3] Merritt MA, Cramer DW (2011). Molecular pathogenesis of endometrial and ovarian cancer. Cancer Biomark.

[4] Malik TY, Chishti U, Aziz AB, Sheikh I (2016). Comparison of risk factors and survival of type 1 and type II endometrial cancers. Pak J Med Sci.

[5] Bian X, Liu R, Meng Y, Xing D, Xu D, Lu Z (2021). Lipid metabolism and cancer. J Exp Med.

[6] Hutt S, Tailor A, Ellis P, Michael A, Butler-Manuel S, Chatterjee J (2019). The role of biomarkers in endometrial cancer and hyperplasia: A literature review. Acta Oncol.

[7] Mutter GL, Monte NM, Neuberg D, Ferenczy A, Eng C (2014). Emergence, involution, and progression to carcinoma of mutant clones in normal endometrial tissues. Cancer Res.

[8] Abd El-Maqsoud NM, El-Gelany S (2009). Differential expression patterns of pten in cyclic, hyperplastic and malignant endometrium: its relation with er, pr and clinicopathological parameters. J Egypt Natl Canc Inst.

[9] Joshi A, Wang H, Jiang G, Douglas W, Chan JS, Korach KS, Ellenson LH (2012). Endometrial tumorigenesis in Pten(+/-) mice is independent of coexistence of estrogen and estrogen receptor α. Am J Pathol.

[10] Bhan A, Hussain I, Ansari KI, Bobzean SAM, Perrotti LI, Mandal SS (2014). Histone methyltransferase ezh2 is transcriptionally induced by estradiol as well as estrogenic endocrine disruptors bisphenol-a and diethylstilbestrol. J Mol Biol.

[11] Nanjappa MK, Mesa AM, Medrano TI, Jefferson WN, DeMayo FJ, Williams CJ (2019). The histone methyltransferase EZH2 is required for normal uterine development and function in mice. Biol Reprod.

[12] Eskander RN, Ji T, Huynh B, Wardeh R, Randall LM, Hoang B (2013). Inhibition of enhancer of zeste homolog 2 (EZH2) expression is associated with decreased tumor cell proliferation, migration, and invasion in endometrial cancer cell lines. Int J Gynecol Cancer.

[13] Yang Q, Nair S, Laknaur A, Ismail N, Diamond MP, Al-Hendy A (2016). The polycomb group protein ezh2 impairs dna damage repair gene expression in human uterine fibroids. Biol Reprod.

[14] Gu Y, Zhang J, Guan H (2017). Expression of EZH2 in endometrial carcinoma and its effects on proliferation and invasion of endometrial carcinoma cells. Oncol Lett.

[15] Jiang P, Jia M, Hu J, Huang Z, Deng Y, Lai L (2020). Prognostic value of ki67 in patients with stage 1-2 endometrial cancer: validation of the cut-off value of ki67 as a predictive factor. Onco Targets Ther.

[16] Ferrandina G, Ranelletti FO, Gallotta V, Martinelli E, Zannoni GF, Gessi M, Scambia G (2005). Expression of cyclooxygenase-2 (COX-2), receptors for estrogen (ER), and progesterone (PR), p53, ki67, and neu protein in endometrial cancer. Gynecol Oncol.

[17] Ghalib Farhood R, Abd Ali Al-Humairi I (2022). Immunohistochemical study of ki-67 in hyperplastic and endometrium carcinoma: a comparative study. Arch Razi Inst.

[18] Ataabadi MS, Bahmanpour S, Yousefinejad S, Alaee S (2023). Blood volatile organic compounds as potential biomarkers for poly cystic ovarian syndrome (PCOS): An animal study in the PCOS rat model. J Steroid Biochem Mol Biol.

[19] Korovesis D, Rubio-Tomás T, Tavernarakis N (2023). Oxidative stress in age-related neurodegenerative diseases: An overview of recent tools and findings. Antioxidants (Basel).

[20] Bolton JL (2002). Quinoids, quinoid radicals, and phenoxyl radicals formed from estrogens and antiestrogens. Toxicology.

[21] Punnonen R, Kudo R, Punnonen K, Hietanen E, Kuoppala T, Kainulainen H (1993). Activities of antioxidant enzymes and lipid peroxidation in endometrial cancer. Eur J Cancer.

[22] Fader AN, Arriba LN, Frasure HE, von Gruenigen VE (2009). Endometrial cancer and obesity: Epidemiology, biomarkers, prevention and survivorship. Gynecol Oncol.

[23] Burgovа EN, Khristidis YI, Kurkov AV, Mikoyan VD, Shekhter AB, Adamyan LV (2019). The inhibiting effect of dinitrosyl iron complexes with thiol-containing ligands on the growth of endometrioid tumours in rats with experimental endometriosis. Cell Biochem Biophys.

[24] Rangel-Zuñiga OA, Cruz-Teno C, Haro C, Quintana-Navarro GM, Camara-Martos F, Perez-Martinez P (2017). Differential menopause- versus aging-induced changes in oxidative stress and circadian rhythm gene markers. Mech Ageing Dev.

[25] Bolouki A, Zal F, Alaee S (2020). Ameliorative effects of quercetin on the preimplantation embryos development in diabetic pregnant mice. J Obstet Gynaecol Res.

[26] Chen M, Lin C, Liu CJC (2015). Efficacy of phytoestrogens for menopausal symptoms: A meta-analysis and systematic review. Climacteric.

[27] Grandi G, Facchinetti F, Melotti C, Sgandurra A (2023). Phyto-progestins for the treatment of abnormal uterine bleeding without organic cause in women at high risk for breast cancer and breast cancer survivors: A prospective, pilot study. Gynecol Endocrinol.

[28] Alshehri MM, Sharifi-Rad J, Herrera-Bravo J, Jara EL, Salazar LA, Kregiel D (2021). Therapeutic potential of isoflavones with an emphasis on daidzein. Oxid Med Cell Longev.

[29] Jenks BH, Iwashita S, Nakagawa Y, Ragland K, Lee J, Carson WH (2012). A pilot study on the effects of S-equol compared to soy isoflavones on menopausal hot flash frequency. J Womens Health (Larchmt).

[30] Unfer V, Casini ML, Costabile L, Mignosa M, Gerli S, Di Renzo GC (2004). Endometrial effects of long-term treatment with phytoestrogens: A randomized, double-blind, placebo-controlled study. Fertil Steril.

[31] Mohammad-Shahi M, Haidari F, Rashidi B, Saei AA, Mahboob S, Rashidi MR (2011). Comparison of the effects of genistein and daidzein with dexamethasone and soy protein on rheumatoid arthritis in rats. Bioimpacts.

[32] Vakili S, Zal F, Mostafavi-Pour Z, Savardashtaki A, Koohpeyma F (2021). Quercetin and vitamin E alleviate ovariectomy-induced osteoporosis by modulating autophagy and apoptosis in rat bone cells. J Cell Physiol.

[33] Yang Y, Zheng X, Li B, Jiang S, Jiang L (2014). Increased activity of osteocyte autophagy in ovariectomized rats and its correlation with oxidative stress status and bone loss. Biochem Biophys Res Commun.

[34] Martínez-Montemayor MM, Otero-Franqui E, Martinez J, De La Mota-Peynado A, Cubano LA, Dharmawardhane S (2010). Individual and combined soy isoflavones exert differential effects on metastatic cancer progression. Clin Exp Metastasis.

[35] Maghool F, Khaksari M (2013). Differences in brain edema and intracranial pressure following traumatic brain injury across the estrous cycle: involvement of female sex steroid hormones. Brain Res.

[36] Jahromi BN, Farrokhnia F, Tanideh N, Kumar PV, Parsanezhad ME, Alaee S (2019). Comparing the effects of glycyrrhiza glabra root extract, a cyclooxygenase-2 inhibitor (celecoxib) and a gonadotropin-releasing hormone analog (diphereline) in a rat model of endometriosis. Int J Fertil Steril.

[37] Tanideh N, Daneshmand F, Karimimanesh M, Mottaghipisheh J, Koohpeyma F, Koohi-Hosseinabadi O (2023). Hydroalcoholic extract of Glycyrrhiza glabra root combined with Linum usitatissimum oil as an alternative for hormone replacement therapy in ovariectomized rats. Heliyon.

[38] Aebi H (1984). Catalase in vitro. Methods Enzymol.

[39] Zal F, Mahdian Z, Zare R, Soghra B, Mostafavi-Pour Z (2014). Combination of vitamin E and folic acid ameliorate oxidative stress and apoptosis in diabetic rat uterus. Int J Vitam Nutr Res.

[40] Bradford MM (1976). A rapid and sensitive method for the quantitation of microgram quantities of protein utilizing the principle of protein-dye binding. Anal Biochem.

[41] Teixeira CP, Simões RS, Santos MA, Calió ML, Soares JM Jr, Simões MJ (2014). Soybean concentrated extract counteracts oxidative stress in the uterus of rats. Climacteric.

[42] Zhao H, Li X, Li N, Liu T, Liu J, Li Z (2014). Long-term resveratrol treatment prevents ovariectomy-induced osteopenia in rats without hyperplastic effects on the uterus. Br J Nutr.

[43] Lindberg MK, Weihua Z, Andersson N, Movérare S, Gao H, Vidal O (2002). Estrogen receptor specificity for the effects of estrogen in ovariectomized mice. J Endocrinol.

[44] Lohrasbi P, Karbalay-Doust S, Mohammad Bagher Tabei S, Azarpira N, Alaee S, Rafiee B, Bahmanpour S (2022). The effects of melatonin and metformin on histological characteristics of the ovary and uterus in letrozole-induced polycystic ovarian syndrome mice: A stereological study. Int J Reprod Biomed.

[45] Ring KL, Mills AM, Modesitt SC (2022). Endometrial hyperplasia. Obstet Gynecol.

[46] Palmer J, Perunovic B, Tidy J (2008). Endometrial hyperplasia. Obstet Gynaecol.

[47] Ishimi Y, Miyaura C, Ohmura M, Onoe Y, Sato T, Uchiyama Y (1999). Selective effects of genistein, a soybean isoflavone, on B-lymphopoiesis and bone loss caused by estrogen deficiency. Endocrinology.

[48] Hewitt SC, Korach KS (2018). Estrogen receptors: New directions in the new millennium. Endocr Rev.

[49] Kim JH, Kim YJ (2015). Effects of genistein in combination with conjugated estrogens on endometrial hyperplasia and metabolic dysfunction in ovariectomized mice. Endocr J.

[50] Zhang Y, Zhou LP, Li XL, Zhao YJ, Ho MX, Qiu ZC (2018). 8-Prenylgenistein, a prenylated genistein derivative, exerted tissue selective osteoprotective effects in ovariectomized mice. Oncotarget.

[51] Hojnik M, Sinreih M, Anko M, Hevir-Kene N, Knific T, Pirš B (2023). The co-expression of estrogen receptors ERα, ERβ, and GPER in endometrial cancer. Int J Mol Sci.

[52] Liu D-Y, He S-J, Jin E-H, Liu S-Q, Tang Y-G, Li S-H, Zhong L-T (2013). Effect of daidzein on production performance and serum antioxidative function in late lactation cows under heat stress. Livest Sci.

[53] Aguilar M, Chen H, Rivera-Colon G, Niu S, Carrick K, Gwin K (2022). Reliable identification of endometrial precancers through combined pax2, β-catenin, and pten immunohistochemistry. Am J Surg Pathol.

[54] Raffone A, Travaglino A, Saccone G, Campanino MR, Mollo A, De Placido G (2019). Loss of PTEN expression as diagnostic marker of endometrial precancer: A systematic review and meta-analysis. Acta Obstet Gynecol Scand.

[55] Mesa AM, Mao J, Nanjappa MK, Medrano TI, Tevosian S, Yu F (2020). Mice lacking uterine enhancer of zeste homolog 2 have transcriptomic changes associated with uterine epithelial proliferation. Physiol Genomics.

[56] Li LT, Jiang G, Chen Q, Zheng JN (2015). Ki67 is a promising molecular target in the diagnosis of cancer (review). Mol Med Rep.

[57] Akbarzadeh-Jahromi M, Jafari F, Parsanezhad ME, Alaee S (2022). Evaluation of supplementation of cryopreservation medium with gallic acid as an antioxidant in quality of post-thaw human spermatozoa. Andrologia.

[58] Yousef MI, Kamel KI, Esmail AM, Baghdadi HH (2004). Anti-oxidant activities and lipid lowering effects of isoflavone in male rabbits. Food Chem Toxicol.

