# Supplementary material for: Comparative effects of estradiol and daidzein on the expression of endometrial cancer-related genes and histopathological parameters in the uterus of ovariectomized rats
Source: Iran J Basic Med Sci. 2026;29(1):145–54. doi: 10.22038/ijbms.2025.89681.19349 (PMC12867098; doi:10.22038/ijbms.2025.89681.19349)
Supplement: Supplementary file 1 — Figure S1-S4 [file ijbms-29-1-145-s001.pdf]

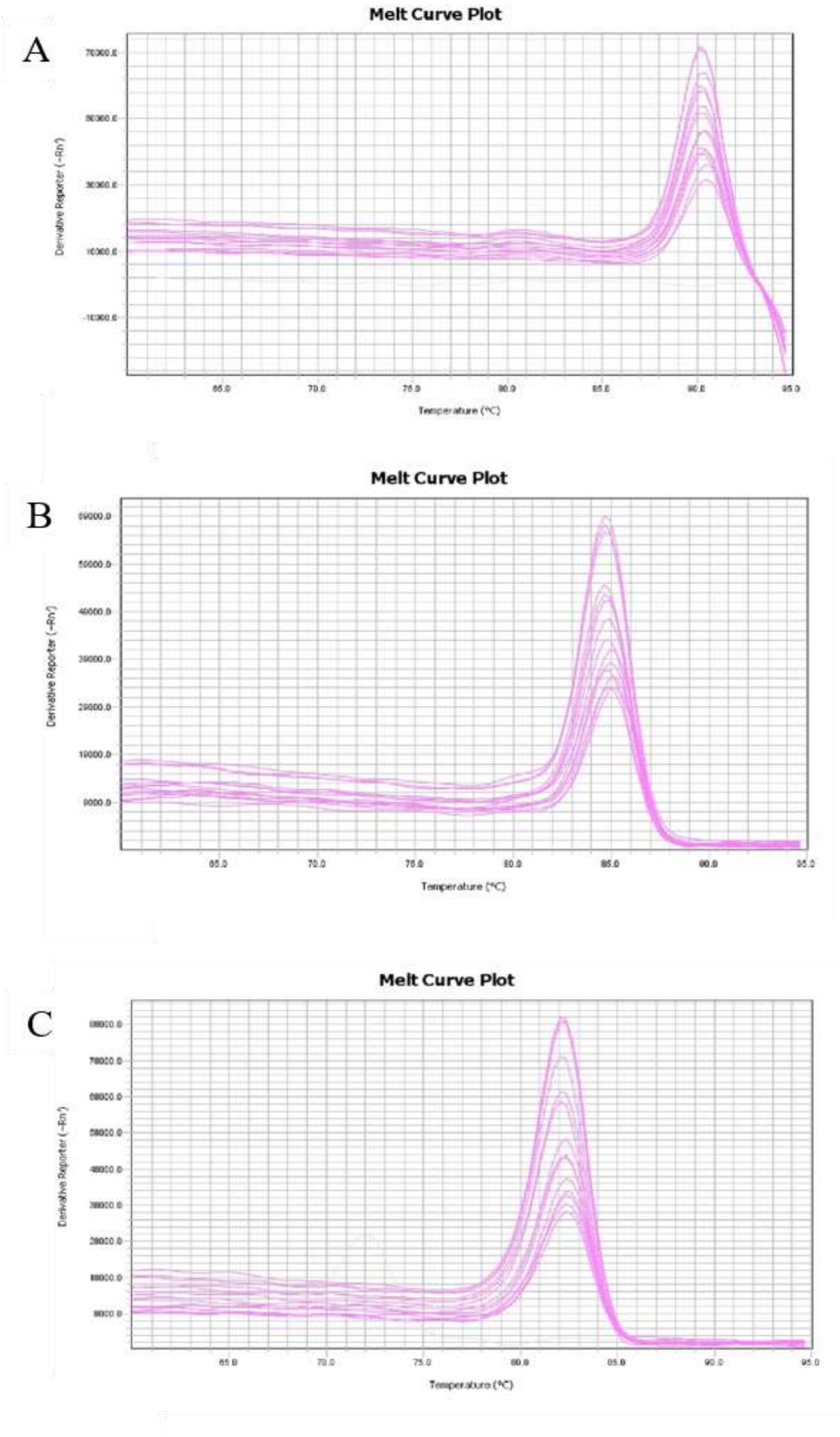

Supplementary Figure S1. Melting curve analyses for the reference gene and estrogen receptors

(A) β-actin (Reference gene), (B) ERα (Esr1), (C) ERβ (Esr2). A single sharp peak for each assay confirms the amplification of a specific product without primer-dimers or non-specific amplification.

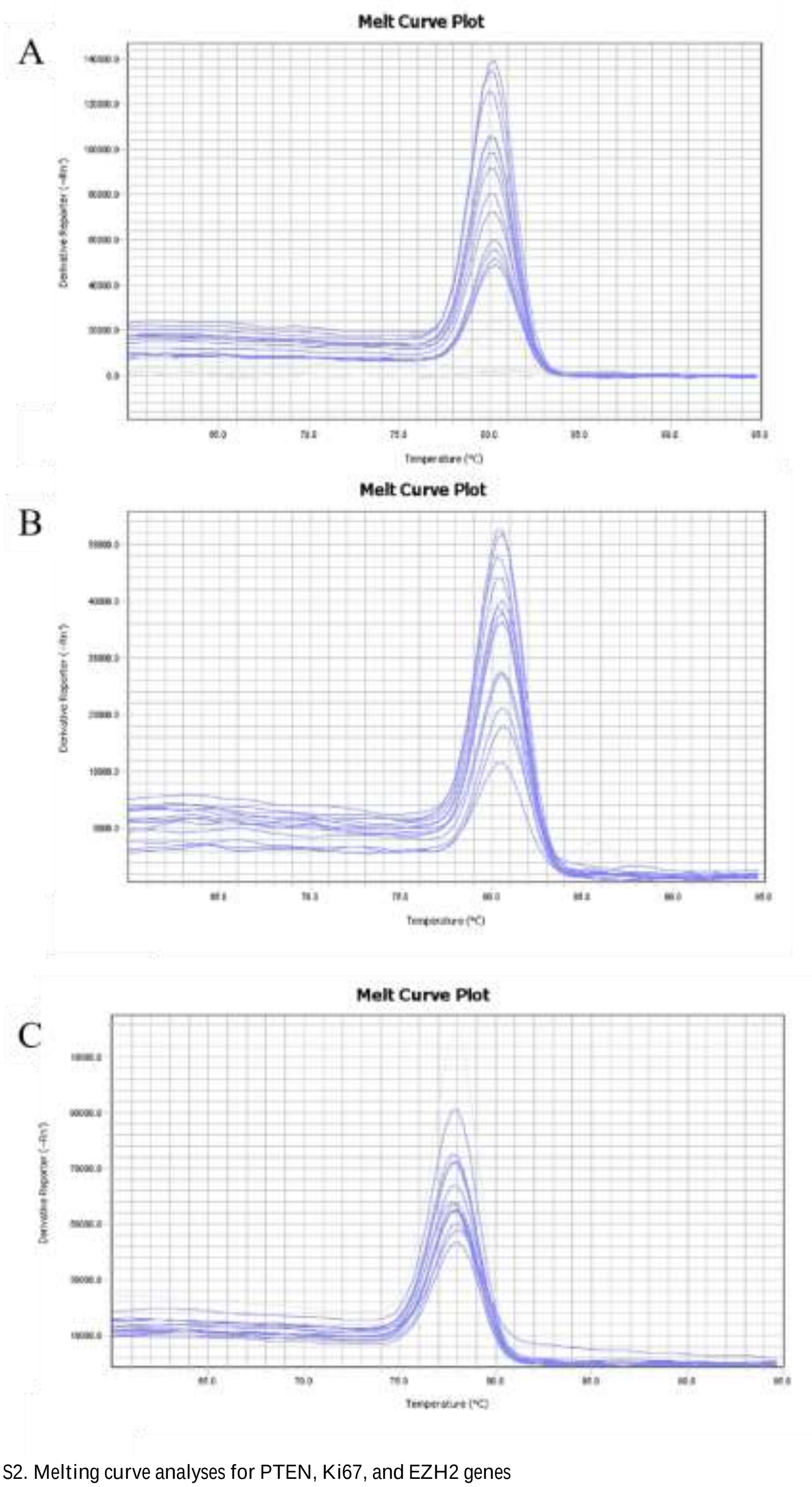

Supplementary Figure S2. Melting curve analyses for PTEN, Ki67, and EZH2 genes

(A) PTEN, (B) Ki67 (Mki67), (C) EZH2. The presence of a single distinct peak for each gene demonstrates the specificity of the primer pairs used in the qRT-PCR assays.

A

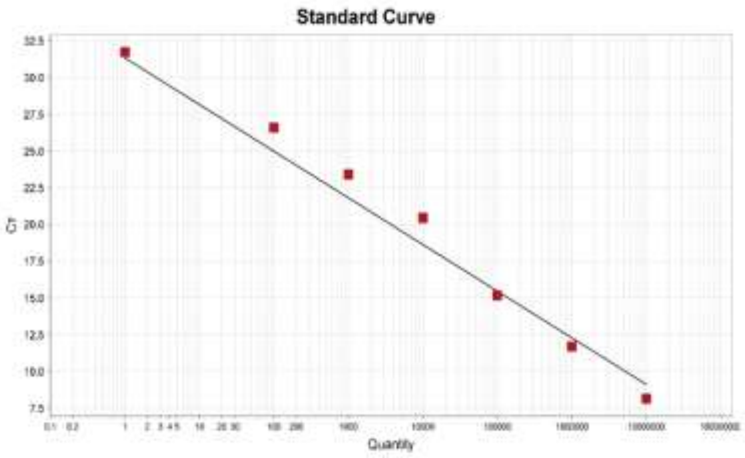

B

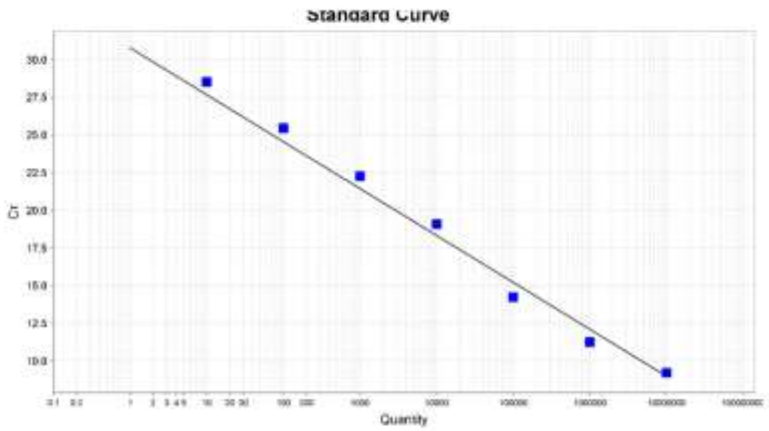

C

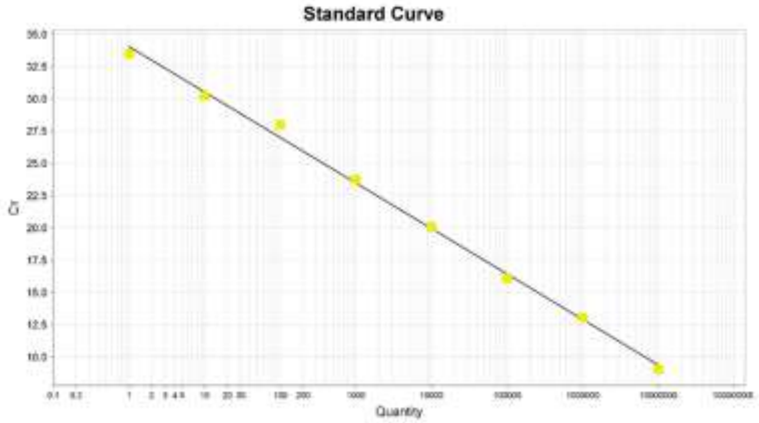

Supplementary Figure S3. Standard curves for the reference gene and estrogen receptors

Serial dilutions of cDNA were used to generate standard curves for (A) β-actin, (B) ERα, and (C) ERβ.

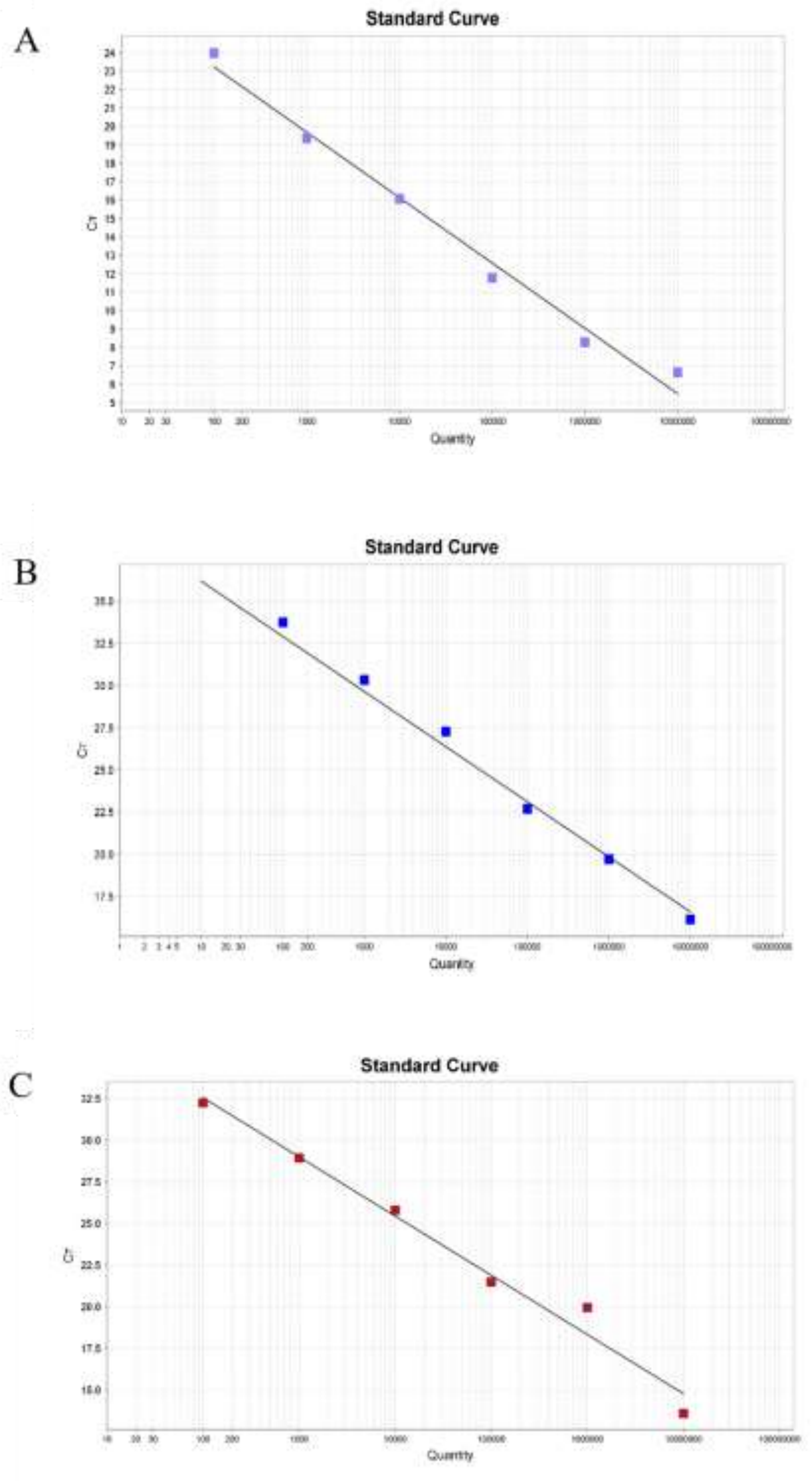

Supplementary Figure S4. Standard curves for PTEN, Ki67, and EZH2 genes

Standard curves generated from serial cDNA dilutions for (A) PTEN, (B) Ki67, and (C) EZH2.
