# Supplementary material for: Comparative effects of estradiol and daidzein on the expression of endometrial cancer-related genes and histopathological parameters in the uterus of ovariectomized rats
Source: Iran J Basic Med Sci. 2026;29(1):145–54. doi: 10.22038/ijbms.2025.89681.19349 (PMC12867098; doi:10.22038/ijbms.2025.89681.19349)
Supplement: Supplementary file 2 — Table S1 [file ijbms-29-1-145-s002.pdf]

Table S1. Primer sequences, amplicon sizes, and amplification efficiencies

Gene Symbol	Forward Primer (5'→3')	Reverse Primer (5'→3')	Amplicon Size (bp)	Efficiency (%)
β-actin	AAGGCCAACCGTGAAAAGAT	ACCAGAGGCATACAGGGAC	102	106
ERα	TGCTCTTGGACAGGAATC	AAGGTGCTGGATAGAAATG	175	102
ERβ	GACCCATTGCCAATCATCGC	CCTCATCCCTGTCCAGAACG	141	92
PTEN	ACGGACTGGTGTAATGATT	GCCTCTGACTGGGAATAG	131	95
Ki67	CTCTCACATCCACCTTAC	GGGTTCTAACTGGTCTTCCTG	78	102
EZH2	GATGACGATGATGATGATGGA	CGAGGTGGGCAAGTTTCT	98	91

Details of the primer pairs used for qRT-PCR analysis. The amplification efficiency for each primer set was calculated from standard curves derived from serial cDNA dilutions.
